# Genetic-deletion of Cyclooxygenase-2 Downstream Prostacyclin Synthase Suppresses Inflammatory Reactions but Facilitates Carcinogenesis, unlike Deletion of Microsomal Prostaglandin E Synthase-1

**DOI:** 10.1038/srep17376

**Published:** 2015-11-27

**Authors:** Yuka Sasaki, Shuhei Kamiyama, Azusa Kamiyama, Konomi Matsumoto, Moe Akatsu, Yoshihito Nakatani, Hiroshi Kuwata, Yukio Ishikawa, Toshiharu Ishii, Chieko Yokoyama, Shuntaro Hara

**Affiliations:** 1 Division of Health Chemistry, Department of Healthcare and Regulatory Sciences, School of Pharmacy, Showa University, Tokyo 142-8555, Japan; 2 Pathology Section, Itabashi Medical Laboratory, Tokyo 174-0051, Japan; 3 Department of Pathology, Saiseikai Yokohama City Tobu Hospital, Yokohama 230-8765, Japan; 4 Kanagawa Institute of Technology, Atsugi 243-0292, Japan

## Abstract

Prostacyclin synthase (PGIS) and microsomal prostaglandin E synthase-1 (mPGES-1) are prostaglandin (PG) terminal synthases that function downstream of inducible cyclooxygenase (COX)-2 in the PGI_2_ and PGE_2_ biosynthetic pathways, respectively. mPGES-1 has been shown to be involved in various COX-2-related diseases such as inflammatory diseases and cancers, but it is not yet known how PGIS is involved in these COX-2-related diseases. Here, to clarify the pathophysiological role of PGIS, we investigated the phenotypes of PGIS and mPGES-1 individual knockout (KO) or double KO (DKO) mice. The results indicate that a thioglycollate-induced exudation of leukocytes into the peritoneal cavity was suppressed by the genetic-deletion of PGIS. In the PGIS KO mice, lipopolysaccharide-primed pain nociception (as assessed by the acetic acid-induced writhing reaction) was also reduced. Both of these reactions were suppressed more effectively in the PGIS/mPGES-1 DKO mice than in the PGIS KO mice. On the other hand, unlike mPGES-1 deficiency (which suppressed azoxymethane-induced colon carcinogenesis), PGIS deficiency up-regulated both aberrant crypt foci formation at the early stage of carcinogenesis and polyp formation at the late stage. These results indicate that PGIS and mPGES-1 cooperatively exacerbate inflammatory reactions but have opposing effects on carcinogenesis, and that PGIS-derived PGI_2_ has anti-carcinogenic effects.

Non-steroidal anti-inflammatory drugs (NSAIDs) exert their anti-inflammatory and anti-tumor effects by reducing the production of prostaglandins (PGs) by inhibiting cyclooxygenase (COX)^1^,^2^,^3^. Of the two COX isozymes COX-1 and COX-2, COX-1 is expressed constitutively in most tissues and is generally responsible for the production of the PGs that control normal physiological functions, whereas COX-2 is induced in response to mitogens, cytokines, and cellular transformation and is related to inflammatory reactions and carcinogenesis. The long-term use of NSAIDs is associated with severe side effects, mainly gastrointestinal injury and renal irritations, apparently due to impaired COX-1-dependent PG biosynthesis. Although COX-2-selective inhibitors show reduced gastrointestinal complications, recent clinical trials indicated a significantly increased cardiovascular risk of these agents^4^. The specific inhibition of COX-2 alters the balance between platelet-derived thromboxane A_2_ (TXA_2_) and endothelium-derived prostacyclin (PGI_2_), leading to increases in the risk of thrombosis due to altered vascular tone. Thus, for the development of novel NSAIDs without adverse side effects, a more selective modulation of PG production appears to be desirable.

PGH_2_, a COX metabolite, is converted to each PG species by species-specific PG terminal synthases. Among the PG terminal synthases, microsomal PGE synthase-1 (mPGES-1) is induced by pro-inflammatory stimuli or mitogens and down-regulated by anti-inflammatory glucocorticoids, as in the case of COX-2, and is functionally coupled with COX-2 in marked preference to COX-1^5^,^6^,^7^,^8^. The induction of mPGES-1 expression has been observed in various conditions and processes in which COX-2-driven PGE_2_ has been implicated, including rheumatoid arthritis and cancer. Other research groups and our group have used mPGES-1 knockout (KO) mice, finding that mPGES-1 plays a critical role in inflammatory reactions and carcinogenesis^7^,^8^,^9^,^10^,^11^. Now, mPGES-1 has gained attention as a novel target for NSAIDs. Several mPGES-1-selective inhibitors were recently developed and shown to suppress inflammatory reactions in experimental animal models^12^,^13^,^14^.

However, not only mPGES-1 but also PGI_2_ synthase (PGIS) functionally couples with COX-2^5^,^15^. In mPGES-1 KO mice, COX-2-derived PGH_2_ is metabolically shunted into PGIS-mediated PGI_2_ production^9^,^11^,^16^. PGIS is highly expressed in vascular endothelial and smooth muscle cells, but a variety of cells other than vascular cells including macrophages also express PGIS^17^,^18^,^19^,^20^. Studies using PGI_2_ receptor (IP)-KO mice have also revealed that PGI_2_ is involved in inflammatory and pain responses as well as the regulation of vascular tone^21^,^22^,^23^,^24^, but the question of how PGIS is involved in COX-2-related diseases such as inflammatory diseases and cancers has not been fully answered.

To reveal the pathophysiological roles of PGIS in inflammatory reaction and carcinogenesis, we here established PGIS and mPGES-1 double knockout (DKO) mice and investigated the phenotypes of PGIS- and mPGES-1-single KO mice and the DKO mice. The results demonstrated that the genetic-deletion of PGIS suppresses inflammatory reactions but facilitates carcinogenesis, unlike mPGES-1 deletion. These findings indicated that understanding the relationships among the PG terminal synthases is crucial to the development of novel NSAIDs without adverse side effects.

## Results

### Establishment of PGIS/mPGES-1 DKO mice and characterization of macrophages derived from KO mice

For the establishment of PGIS(−/−)mPGES-1(−/−) mice (DKO mice), PGIS(−/−) mice were crossed with mPGES-1(−/−) mice to generate PGIS(+/−)mPGES-1(+/−) mice, and then these heterozygous mice were intercrossed. In addition, PGIS(−/−)mPGES-1(−/−) mice were crossed with PGIS(+/−)mPGES-1 (+/−) mice. Among the 233 progenies of these crosses, 73 (31.3%) were PGIS(+/−)mPGES-1(+/−) (as control mice in the following study), 59 (25.3%) were PGIS(−/−)mPGES-1(+/−) (as PGIS KO mice), 55 (23.6%) were PGIS(+/−)mPGES-1(−/−) (as mPGES-1 KO mice), and 46 (19.7%) were PGIS(−/−)mPGES-1(−/−) (as PGIS/mPGES-1 DKO mice). The number of DKO mice was slightly less than the expected Mendelian ratio, but all four genotypes of mice were born.

**Figure 1:**
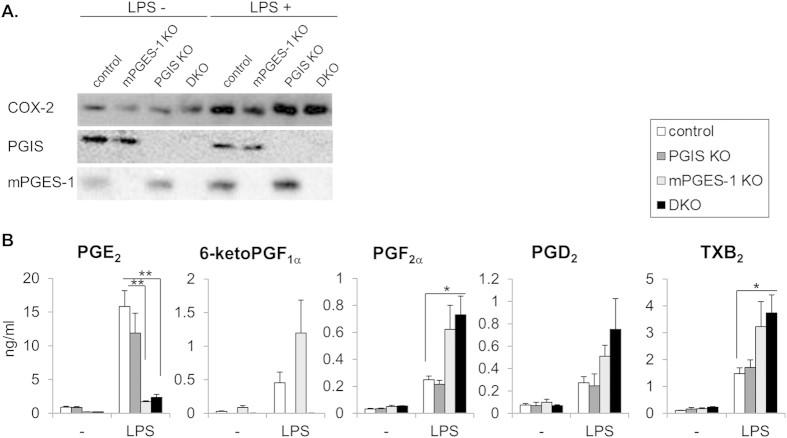
Expression of COX-2, PGIS and mPGES-1 and production of prostanoids in peritoneal MΦs prepared from PGIS and/or mPGES-1 KO mice. (**A**) Immunoblot analysis of COX-2, PGIS and mPGES-1 expression in the control, mPGES-1 KO, PGIS KO, and PGIS/mPGES-1 DKO MΦs. Peritoneal MΦs were prepared from thioglycollate-treated mice, and then incubated with or without LPS for 24 hours. Cell lysates were prepared and subjected to immunoblot analysis. (**B**) Amounts of prostanoids in culture medium from these MΦs. Culture media from LPS-treated MΦs were subjected to a lipidomics analysis using LC-ESI-MS. Results are mean ± SEM (n = 3–8). *P < 0.05 vs. control.

For the determination of whether PGIS and mPGES-1 were indeed knocked out in the null mice, the protein levels in thioglycollate-induced peritoneal macrophages (MΦs) prepared from the four genotypes of mice were analyzed by Western blotting (Fig. 1A). In the control and PGIS-deficient MΦs, mPGES-1 protein was detected in normal culture conditions as was COX-2, and mPGES-1 was up-regulated by the lipopolysaccharide (LPS) treatment. On the other hand, PGIS protein was detected in the control and mPGES-1-deficient MΦs. Neither mPGES-1 nor PGIS was detected in MΦs prepared from PGIS/mPGES-1 DKO mice. Since it has been reported that LPS-stimulated PGE_2_ production was markedly suppressed and the production of other prostanoids was increased in MΦs derived from mPGES-1 KO mice relative to those derived from wild-type (WT) mice^9^,^16^,^25^,^26^, we further measured prostanoids in culture medium from four genotypes of MΦs. As reported previously, mPGES-1 deficiency decreased the PGE_2_ levels but conversely increased the levels of PGs other than PGE_2_ including 6-ketoPGF_1α_, a PGI_2_ metabolite, in culture medium (Fig. 1B), indicating that COX-2-derived PGH_2_ is metabolically shunted into the other prostanoid synthetic pathway in mPGES-1-deficient MΦs.

In the DKO MΦs, a more marked shunting reaction into the other prostanoids was observed. These shunting phenomena were not observed in PGIS-deficient MΦs, in whose culture medium the levels of prostanoids including PGE_2_ were similar to those in the control MΦs. These results suggested that the intracellular shunting of COX-2-derived PGH_2_ might have a single direction in mouse peritoneal MΦs. In MΦs, mPGES-1 might be not able to metabolize PGH_2_, which should be supplied to PGIS from COX-2.

**Figure 2:**
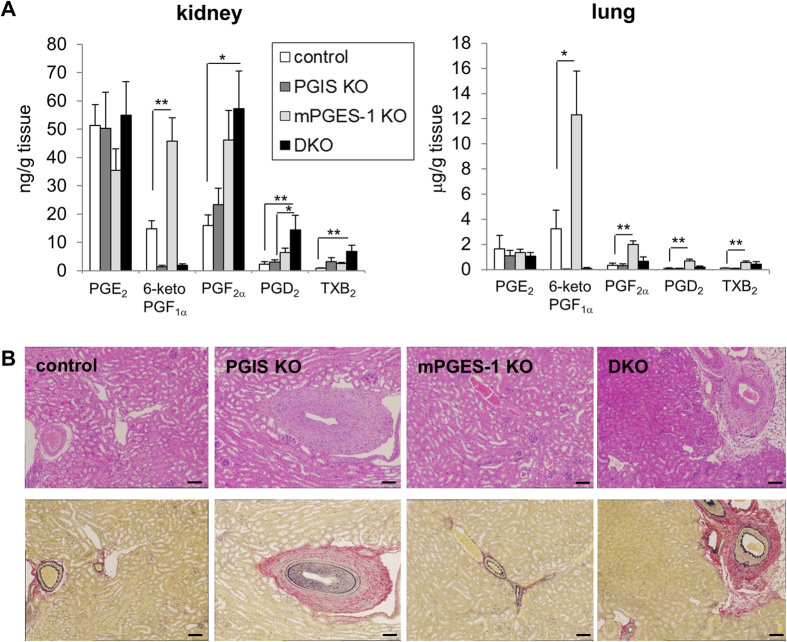
Prostanoid levels in kidney and lungs and histological features of kidney prepared from PGIS and/or mPGES-1 KO mice. (**A**) Prostanoid levels in kidneys and lungs. Kidneys and lungs were resected from mice and then homogenized. Their lipids were extracted and subjected to a lipidomics analysis by LC-ESI-MS. Results are mean ± SEM (n = 3–6). *P < 0.05 and **P < 0.01 vs. control. (**B**) Histological features of kidneys. Sections of kidneys were stained with hematoxylin and eosin (H&E, upper panel) or Elastica van Gieson stain (lower panel). Bar: 100 μm.

It has been shown that PGIS and mPGES-1 are expressed in mouse kidney and lung even under basal conditions^9^,^16^,^18^,^19^,^27^. We next measured the prostanoid levels in kidneys and lungs prepared from the four genotypes of mice and found that these two organs also showed a shunting reaction similar to that of the MΦs (Fig. 2A). Metabolic shunting of PGH_2_ was observed in the mPGES-1-deficient mice but not in PGIS-deficient mice. We further found that the DKO mice developed renal disorders with arterial sclerosis and hypertrophy of vessels (Fig. 2B), as did the PGIS single-KO mice^28^. These results suggested that renal disorders observed in PGIS-deficient mice might be induced by a breakdown of PGI_2_ levels but not by shunting into prostanoids other than PGI_2_.

### PGIS is involved in inflammatory reactions

**Figure 3:**
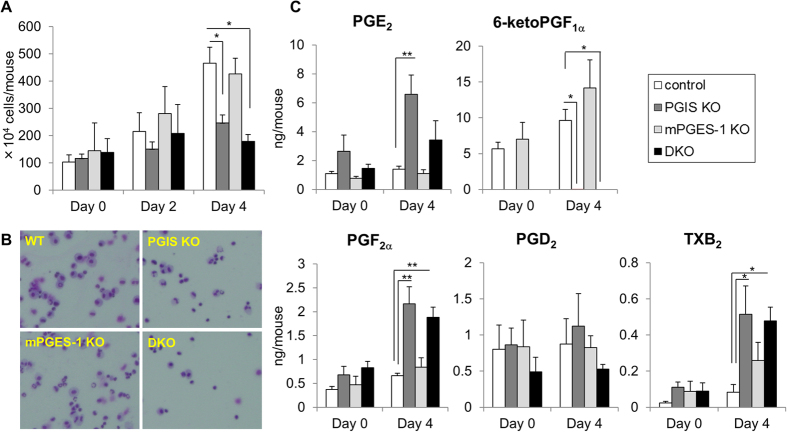
Involvement of PGIS and mPGES-1 in thioglycollate-induced peritonitis. Number and morphology of exudate leukocytes (**A,B**) and prostanoid levels in peritoneal fluids (**C**) from thioglycollate-treated PGIS and/or mPGES-1 KO mice. (**A**) Peritoneal exudate cells and fluids were collected from mice on day 0, 2 or 4 after the injection of thioglycollate. The peritoneal cells were washed and then counted. (**B**) Representative Giemsa staining of cytocentrifuge preparations of the exudate leukocytes on day 4. (**C**) The peritoneal fluids were subjected to a lipidomics analysis by LC-ESI-MS. Results are mean ± SEM (n = 3–6). *P < 0.05 and **P < 0.01 vs. control.

Studies using KO mice have revealed that IP and PGE_2_ receptor subtypes EP2 and EP4 are involved in inflammatory reactions including swelling and pain^21^,^22^,^23^,^24^. Thus, to examine the effects of PGIS and mPGES-1 deficiency on inflammatory reaction, we next counted the number of exudate leukocytes and measured the levels of prostanoids in the peritoneal fluids prepared from the four genotypes of thioglycollate-treated mice. As shown in Fig. 3, in the control mice, the peritoneal leukocyte number was increased steadily from day 2 to 4 after the injection of thioglycollate, accompanied by an increase in 6-ketoPGF_1α_ levels. PGIS deficiency did not significantly affect the number of exudate leukocytes on day 2, but significantly decreased their number on day 4 compared to the control mice. Our morphological analysis revealed that among leukocytes, the exudation of MΦs was especially suppressed by PGIS deficiency (Supplementary Table 1). In the PGIS KO mice, 6-ketoPGF_1α_ was not detected in the peritoneal fluid. Unlike the PGIS-deficient MΦs, PGE_2_ and the other prostanoid levels in the PGIS KO mice were higher than those in the control mice. These results indicated that PGIS-derived PGI_2_ might play an important role in the exudation of MΦs at the late stage of inflammatory reaction.

Moreover, the migration of MΦs into the peritoneal fluid was not affected by mPGES-1 deficiency but was suppressed more effectively in the PGIS/mPGES-1 DKO mice than in the PGIS KO mice. mPGES-1-derived PGE_2_ might contribute to the exudation of MΦs, but its contribution might be smaller than that of PGIS-derived PGI_2_.

**Figure 4:**
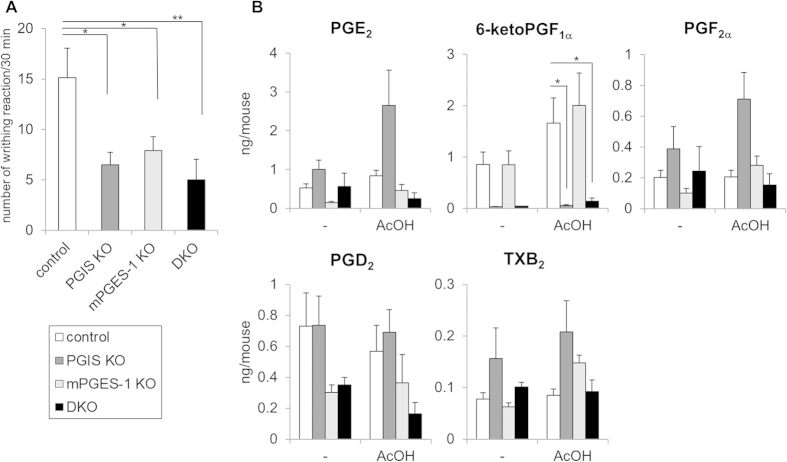
Involvement of PGIS and mPGES-1 in the LPS-primed acetic acid-induced writhing reaction. Number of writhing reactions (**A**) and prostanoid levels in peritoneal fluids (**B**) from LPS-primed acetic acid-treated PGIS and/or mPGES-1 KO mice. (**A**) The writhing reaction was induced in mice by an injection of acetic acid 18 hours after LPS treatment. The number of writhing responses was counted for 30 min after the injection of acetic acid. (**B**) Peritoneal fluids were collected from mice 10 min after the injection of acetic acid (+) or saline (−) and then subjected to a lipidomics analysis by LC-ESI-MS. Results are mean ± SEM (n = 6–10). *P < 0.05 and **P < 0.01 vs. control.

We further investigated the effect of PGIS deficiency on inflammatory pain hypersensitivity, as assessed by the LPS-primed acetic acid-induced writhing reaction. The LPS pretreatment induced the expressions of COX-2 and mPGES-1 and then enhanced the writhing reaction. As we previously reported^9^, the injection of acetic acid into the peritoneum of mice induced a stretching behavior, that peaked at 5–10 min and then declined gradually over 30 min. The writhing reaction was reduced in both the PGIS KO and mPGES-1 KO mice, and it was suppressed more effectively in the PGIS/mPGES-1 DKO mice compared to the PGIS KO and mPGES-1 KO mice (Fig. 4A). In the PGIS KO mice, 6-ketoPGF_1α_ was not observed but the PGE_2_ levels were markedly increased compared to the control mice, although a similar shunting phenomenon was not observed in the mPGES-1 KO mice (Fig. 4B). In the PGIS/mPGES-1 DKO mice, the levels of both 6-ketoPGF_1α_ and PGE_2_ were reduced. These results indicate that PGIS-derived PGI_2_ facilitates inflammatory pain hypersensitivity in a coordinated manner with mPGES-1-derived PGE_2_.

### PGIS is involved in chemically-induced carcinogenesis

**Figure 5:**
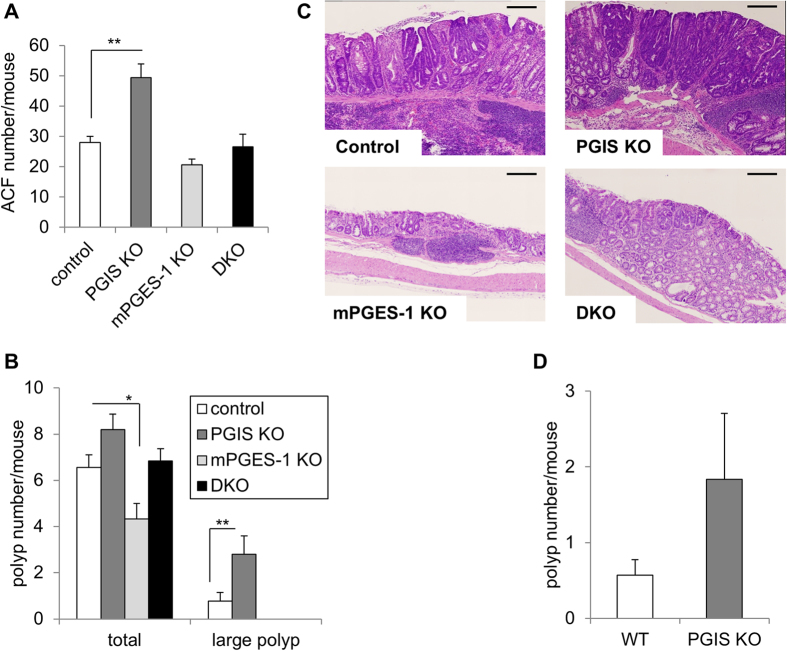
Involvement of PGIS and mPGES-1 in AOM-induced colon carcinogenesis. Numbers of ACFs (**A**) and polyps (**B,D**) in the colon tissues from AOM-treated PGIS and/or mPGES-1 KO mice. (**A**) PGIS and/or mPGES-1 KO mice on a Balb/c background were intraperitoneally injected with AOM once a week for 6 weeks and killed 6 weeks after the last injection. The number of ACFs per mouse in the colon tissues is shown. Results are mean ± SEM (n = 4–5). **P < 0.01 vs. control. (**B**) PGIS and/or mPGES-1 KO mice on a Balb/c background were killed 20 weeks after the last injection of AOM. The numbers of total polyps and large (>2 mm) polyps per mouse in the colon tissues are shown. Results are mean ± SEM (n = 5–9). *P < 0.05 and **P < 0.01 vs. control. (**C**) Histological features of colon tissues. Representative H&E staining of the colon tissues from AOM-treated mice. Bar: 100 μm. (**D**) PGIS KO and littermate WT mice on a C57BL/6 background were intraperitoneally injected with AOM and killed 20 weeks after the last injection. The numbers of total polyps and large (>2 mm) polyps per mouse in the colon tissues are shown. Results are mean ± SEM (n = 6–9). *P < 0.05 and **P < 0.01 vs. control.

We next injected azoxymethane (AOM) intraperitoneally into these four genotypes of mice once a week for 6 weeks to induce colon carcinogenesis. To examine the involvement of PGIS in an early phase of carcinogenesis, we first killed animals 6 weeks after the last injection, and evaluated the preneoplastic aberrant crypt foci (ACF) formation (Fig. 5A). The results indicated that PGIS deficiency did not induce spontaneous colon carcinogenesis but significantly increased the number of ACF, whereas mPGES-1 deficiency decreased the ACF number, as described previously^11^. In the PGIS/mPGES-1 DKO mice, the ACF number was similar to that in the control mice. These results indicate that the genetic-deletion of PGIS facilitates tumor propagation even though it is not sufficient for tumor initiation.

At 20 weeks after the last injection, the AOM administration induced the development of multiple tumors in the control colons, whereas mPGES-1 deficiency suppressed the colon carcinogenesis as described previously^11^. In contrast to mPGES-1 deficiency, PGIS deficiency tended to increase the polyp numbers (Fig. 5B). In addition, the number of large polyps was significantly increased in the PGIS KO mice. As shown in Fig. 5C, histologically, adenocarcinomas were observed in the control, PGIS KO and DKO mice, but only adenomas were observed in the mPGES-1 KO mice. Among them, the adenocarcinomas in the PGIS KO mice were considerably larger than those in the control mice. Balb/c background mice were used for these analyses, because it was shown that Balb/c mice are more sensitive to AOM-induced colon carcinogenesis than other strain mice^29^. We also found that the number of colon polyps (and the number of large polyps in particular) was increased in the PGIS KO mice compared to the wild type (WT) mice even in the C57BL/6 mouse strain, which has been well characterized as being highly resistant to colon tumor induction by AOM^29^ (Fig. 5D). These results indicated that the genetic-deletion of PGIS exacerbates chemically induced colon carcinogenesis.

**Figure 6:**
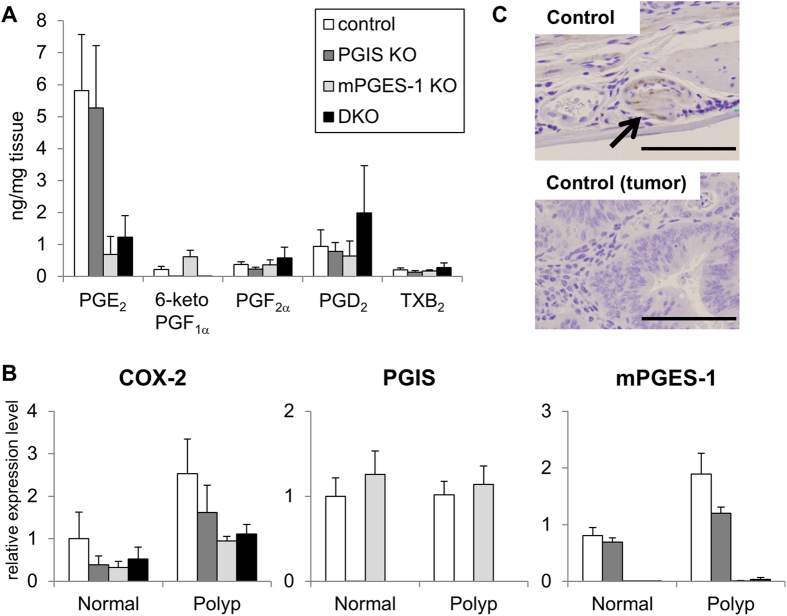
Prostanoid levels and expression of COX-2, PGIS and mPGES-1 in colon tissues prepared from AOM-treated PGIS and/or mPGES-1 KO mice. PGIS and/or mPGES-1 KO mice on a Balb/c background were intraperitoneally injected with AOM once a week for 6 weeks and killed 20 weeks after the last injection. Tissues from each colon tissue were divided into normal and tumor tissues. (**A**) Prostanoid levels in colon tumor tissues. Lipids were extracted from colon tumor tissues of AOM-treated mice and subjected to a lipidomics analysis by LC-ESI-MS. Results are mean ± SEM (n = 4–7). (**B**) Expression of COX-2, PGIS and mPGES-1 mRNAs in colon tissues. COX-2, PGIS and mPGES-1 mRNA levels in normal or tumor colon tissues from AOM-treated mice were assessed by real-time RT-PCR. Results are mean ± SEM (n = 3–5). (**C**) Representative PGIS-immunostaining of the normal or tumor colon tissues from AOM-treated WT mice. Bar: 100 μm.

We next analyzed the levels of prostanoids in these colon polyp tissues (Fig. 6A). As expected, in polyp tissues, mPGES-1 deficiency decreased the PGE_2_ levels, and 6-ketoPGF_1α_ was not detected in the PGIS KO mice or DKO mice. The levels of PGs other than PGE_2_ including 6-ketoPGF_1α_ in the polyp tissues of the mPGES-1 KO mice were higher than those in the control mice, but a similar shunting phenomenon was not observed in the colon polyp tissues of the PGIS KO mice. In the PGIS KO mice, the level of PGE_2_ was similar to that in the control mice. We analyzed the expression levels of COX-2, PGIS and mPGES-1 in colon tissues by quantitative RT-PCR. As shown in Fig. 6B, the levels of both COX-2 and mPGES-1 mRNA in polyp tissues were substantially higher than those in normal tissues of the colon. On the other hand, the PGIS mRNA level in the polyps was similar to that in the normal tissues. In our immunohistochemical analysis, the positive immunostaining signal of PGIS was observed only in blood vessels, not in tumor cells or tumor stromal cells (Fig. 6C). These results suggested that PGIS in host-derived vascular cells might be involved in carcinogenesis and that the breakdown of PGI_2_ in host-derived vascular cells might lead to an exacerbation of colon tumors in PGIS KO mice.

## Discussion

We here established mice that are doubly deficient for PGIS and mPGES-1, both of which are preferentially coupled with COX-2 as their upstream enzymes, and we then investigated the phenotypes of these mice. PGIS/mPGES-1 DKO mice were born. However as well as COX-2 KO mice^30^, when heterozygous KO mice were intercrossed, the number of DKO mice was slightly less than the expected Mendelian ratio. We also observed the development of renal disorders in the DKO mice (Fig. 2B) as well as in the COX-2 KO^30^ and PGIS KO mice^28^. These results suggested that renal disorders might be induced by a breakdown of COX-2/PGIS-derived PGI_2_ levels.

The inflammatory reactions and inflammatory pain hypersensitivity were significantly suppressed in the PGIS KO mice, and they tended to be suppressed more effectively in the PGIS/mPGES-1 DKO mice than in the PGIS KO mice (Figs 3A and 4A). It has been shown that IP as well as EP receptors and mPGES-1 are involved in inflammatory reactions including swelling and pain^21^,^22^,^23^,^24^. The present results indicated that PGIS-derived PGI_2_ acts on IP at inflamed sites and exacerbates inflammation together with mPGES-1-derived PGE_2_. It is noteworthy that PGIS deficiency increased the PGE_2_ levels in inflammatory exudates but mPGES-1 deficiency did not affect the production of PGs other than PGE_2_ in inflammatory exudates (Figs 3B and 4B).

The shunting pattern of PGH_2_ observed in the exudates from KO mice was different from that in KO MΦs (Fig. 1B). At inflamed sites, as well as MΦs, several types of cells (including other leukocytes, vascular cells and stromal cells) are able to produce PGs. In the present case, MΦs might contribute less to PG production. Otherwise, the intracellular transport of PGH_2_ from PGIS-deficient MΦs to the other inflammatory cells might occur. For the development of inhibitors specific for each PG terminal synthase as anti-inflammatory drugs, it is necessary to determine which types of cells are involved in the process of the target illnesses. Here, we found that mPGES-1 deficiency suppressed the acetic acid-induced pain response but did not affect the thioglycollate-induced leukocyte exudation (Figs 3A and 4A). Unlike COX-2, mPGES-1 might have only limited involvement in certain types of inflammatory reactions.

As shown in Fig. 5, the deletions of PGIS and mPGES-1 showed opposite effects on the chemically-induced colon carcinogenesis. mPGES-1 deficiency suppressed the AOM-induced ACF and polyp formation, but PGIS deficiency enhanced both of them. Keith *et al.* reported that in a smoke-exposure model, pulmonary-specific PGIS-overexpressing mice were chemoprotected from developing lung tumors^31^. Together these findings indicate that PGIS-derived PGI_2_ functions as an anti-carcinogenic agent in several types of cancers. In colon tumor tissues, PGIS was expressed in blood vessels (Fig. 6C) and mPGES-1 was expressed in tumor cells and tumor stromal cells^11^. When tumor and host-associated COX-2/mPGES-1-derived PGE_2_ increase and then exceed the anti-carcinogenic actions of vascular COX-2/PGIS-derived PGI_2_, a tumor might begin to progress. As described previously^11^, the ablation of mPGES-1 resulted not only in the suppression of carcinogenic PGE_2_ production but also in the enhancement of anti-carcinogenic PGI_2_ production. An mPGES-1-specific inhibitor is expected to be a more effective anti-carcinogenic agent than a COX-2-specific inhibitor.

A variable-number tandem repeat polymorphism was detected in the promoter region of human PGIS gene that is associated with promoter activity^32^. Poole *et al.* reported that the PGIS promoter polymorphism might affect the risk of the development of colorectal polyps^33^. They showed that having fewer than six repeats on both PGIS alleles was associated with an increased risk of adenoma development compared with the WT genotype, although having more than six repeats reduced the risk. The PGIS promoter polymorphism may be a predictive marker for the risk of colorectal polyps. It is also noteworthy that IP deficiency did not affect the AOM-induced colonic ACF formation^34^. These results suggested that PGI_2_ might suppress colon carcinogenesis through an unknown receptor other than IP.

A candidate target for the anti-carcinogenic action of PGI_2_ is peroxisome proliferator-activated receptor δ (PPARδ). Gupta *et al.* showed that endogenously synthesized PGI_2_ could serve as a ligand for PPARδ^35^. It was also reported that PPARδ deficiency enhanced AOM-induced polyp formation^36^. PGIS-derived PGI_2_ might act on PPARδ and then suppress carcinogenesis. On the other hand, Zuo *et al.* reported that PPARδ deficiency conversely inhibited colon tumorigenesis in mice^37^. Further studies are needed to clarify the involvement of PGI_2_/PPARδ in colon carcinogenesis.

In conclusion, our present findings demonstrate that PGIS promotes inflammatory reactions cooperatively with mPGES-1 but attenuates carcinogenesis, in contrast to mPGES-1. Both PGIS and mPGES-1 functionally couple with COX-2 as their upstream enzymes^5^,^15^. Toward the development of mPGES-1-specific inhibitors as novel NSAIDs without adverse side effects, the involvement of PGIS in target illnesses should be fully elucidated.

## Methods

### Animals

All animal experiments were performed in accordance with protocols approved by the Institutional Animal Care and Use Committees of Showa University, in accordance with the Standards Relating to the Care and Management of Experimental Animals in Japan. Balb/c and C57BL/6 mice were purchased from Saitama Experimental Animals Supply Co. (Saitama, Japan). mPGES-1 KO mice and PGIS KO mice on a C57BL/6 × 129/SvJ background were described previously^9^,^28^. In a colon carcinogenesis model, we used these KO mice, backcrossed 3 times onto Balb/c background^11^. For the establishment of PGIS/mPGES-1 DKO mice, PGIS(−/−) mice were crossed with mPGES-1(−/−) mice to generate PGIS(+/−)mPGES-1(+/−) mice, and then we intercrossed these heterozygous mice. We also crossed PGIS(−/−)mPGES-1(−/−) mice with PGIS(+/−)mPGES-1(+/−) mice to generate PGIS(+/−)mPGES-1(+/−) (as control mice in this study), PGIS(−/−)mPGES-1(+/−) (as PGIS KO mice), PGIS(+/−)mPGES-1(−/−) (as mPGES-1 KO mice), and PGIS(−/−)mPGES-1(−/−) (as DKO mice). These mice were housed in microisolator cages in a pathogen-free barrier facility. All mice were 6–10 weeks old when used for the described experiments.

### Induction of peritonitis and preparation and activation of peritoneal MΦs

Thioglycollate medium (Becton Dickinson, Sparks, MD) (1 mL/20 g of body weight) was intraperitoneally injected into mice, and peritoneal exudate cells and fluids were collected on day 2 and 4 by washing the cavity with 8 mL of PBS as described previously^9^. Cell number was determined by Trypan Blue exclusion. Cytocentrifuge preparations were Giemsa-stained, and cell subsets were identified and counted. For preparation of MΦs, the peritoneal cells were washed, counted and then seeded into 12-well plates (Iwaki Glass, Tokyo, Japan) at a cell density of 10^6^ cells/mL in 1 mL of RPMI medium (Nissui, Tokyo, Japan) supplemented with 10% (v/v) fetal calf serum. After incubation for 2 hours in a CO_2_ incubator, the supernatants and non-adherent cells were removed. More than 90% of adherent cells were peritoneal MΦs. The cells were then incubated with or without 10 μg/mL LPS from *Escherichia coli* O111:B4 (Sigma, St. Louis, MO) in medium containing 2% serum for 24 hours. The culture media were taken for measurements of prostanoids.

### Acetic acid writhing reaction

The acetic acid writhing reaction was induced in mice by an intraperitoneal injection of 0.9% (v/v) acetic acid solution into mice at a dose of 5 mL/kg, as described previously^9^,^21^. For the induction of COX-2, LPS (10 μg/0.1 mL of saline/mouse) was given intraperitoneally 18 hours before the injection of acetic acid solution. The number of writhing responses was counted every 5 min. For the measurement of prostanoids, mice were sacrificed 10 min after the acetic acid injection, and their peritoneal cavities were washed with 5 mL of PBS.

### Induction of colonic tumors by AOM treatment

Mice were intraperitoneally injected with AOM at a dose of 10 mg/kg body weight once a week for 6 weeks as described previously^11^, and then killed 6 or 20 weeks after the last injection of AOM. After laparotomy, the entire colons were dissected and then macroscopically divided into normal-appearing tissues and polyps as normal and tumor tissues, respectively. For the microscopic analysis, the dissected colons were filled with 10% neutral-buffered formalin and then opened longitudinally from the anus to the cecum. For the analysis of ACF formation, mice were killed 6 weeks after the last injection of AOM, and each colon was stained with 0.2% methylene blue in PBS. Colon tissues were scored under a light microscope for the number of ACFs or polyps per colon.

### Immunoblot analysis

Aliquots of samples were subjected to SDS-PAGE using a 10% gel (for COX-2 and PGIS) or 15% gel (for mPGES-1) under reducing conditions. The separated proteins were electroblotted onto nitrocellulose membranes (Schleicher & Schuell, Hahnenstr, Germany) with a semidry blotter (Bio-Rad Laboratories, Hercules, CA). After blocking with 5% (w/v) skim milk in Tris-buffered saline (TBS) (pH 7.4) containing 0.05% (v/v) Tween-20 (TBS-Tween), the membranes were probed with the respective antibodies for 1.5 h (1:5000 dilution for anti-COX-2 antibodies (Santa Cruz Biotechnology, Dallas, TX), 1:2500 dilution for anti-mPGES-1 (Cayman Chemical, Ann Arbor, MI), anti-PGIS (Genway Biotech, SanDiego, CA) and anti-β-actin antibodies (Sigma, St. Louis, MO) in TBS-Tween). They were then incubated with horseradish peroxidase-conjugated anti-rabbit and anti-mouse immunoglobulin G antibodies (1:5000 dilution in TBS-Tween) for 1 h, and visualized with the ECL western blot system (Perkin-Elmer Life Sciences, Boston, MA), as described previously^6^,^9^.

### Histological staining

After surgery, sections of mouse kidneys were placed in phosphate-buffered formaldehyde overnight, then stored in ethanol and embedded in paraffin. Cross-sections were stained with hematoxylin and eosin and Elastica van Gieson.

### Immunohisotochemical analysis

Immunohistochemistry of the tissue sections was performed as described previously^9^. Briefly, the tissue sections were incubated for 15 min with Target Retrieval Solution (DAKO Japan, Kyoto, Japan) , incubated for 10 min with 3% (v/v) H_2_O_2_, washed three times with TBS for 5 min each, incubated with 5% (w/v) skim milk for 30 min, washed three times with TBS-Tween for 5 min each and incubated for 1 h with anti-PGIS antibody (Genway Biotech, SanDiego, CA) in TBS (1:100 dilution). The sections were then treated with the Envision staining kit (DAKO Japan), followed by counterstaining with hematoxylin.

### RT-PCR analysis

Total RNA was extracted from colon mucosa by homogenization in TRIzol reagent. Two micrograms of RNA from each sample were subjected to a reverse transcription (RT) reaction using the High-Capacity cDNA Reverse Transcription Kit (Applied Biosystems, Carlsbad, CA). A SYBR Green-based protocol and real-time PCR detection system (Applied Biosystems) were used to detect mRNA levels. RNA amounts were normalized against the18S rRNA level.

### Measurement of prostanoids by liquid chromatography-electrospray ionization-mass spectrometry (LC-ESI-MS)

For the extraction of prostanoids from the peritoneal fluids or culture media, an internal standard (50 pg of PGB_2_) was added to medium (500 μg), and then the medium was acidified by the addition of 100 μL of 0.2% (v/v) formic acid followed by 500 μL of ethyl acetate. The samples were mixed and centrifuged at 20,000 *g* for 10 min. The organic layer was retrieved and evaporated to dryness with a vacuum evaporator. Samples were resuspended in 100 μL of mobile phase A (water/acetonitrile/formic acid [63:37:0.02, v/v/v]) and injected into a LC-ESI-MS system. For the extraction of prostanoids from the tissues, the snap-frozen tissues were homogenized at 4 °C in SET buffer using a bead crusher (uT-01, TAITEC, Saitama, Japan), and then the homogenates were centrifuged at 3000 *g* for 5 min at 4 °C. Supernatants were isolated and adjusted to pH 3.0 with 1 M HCl. An internal standard (50 pg of PGB_2_) was added to the samples, and then the samples were passed through a Sep-Pak C18 cartridge (Waters, Milford, MA). The retained PGs were eluted with 3 mL of ethyl acetate/methanol (9:1 [v/v]). The sample solvents were evaporated, and then the PGs were resuspended in 100 μL of mobile phase A and injected into the LC-ESI-MS system.

All mass spectrometric analyses were performed using a Prominence HPLC system (Shimadzu, Kyoto, Japan) equipped with a linear ion trap quadrupole mass spectrometer (QTRAP5500, AB Sciex, Framingham, MA, ), as described previously^38^. Briefly, prostanoids were separated by reverse-phase LC on a TSKgel ODS-100 V column (2.0 × 150 mm inner dia, 5-μm particle, Tohso, Tokyo, Japan) at a flow rate of 300 μL/min at 30 °C. The column was equilibrated in mobile phase A (water/acetonitrile/formic acid [63:37:0.02, v/v/v]). Samples (10 μL) were injected using a 50-μL injection loop and eluted with a linear gradient from 0% to 20% mobile phase B (acetonitrile/isopropanol [1:1, v/v]) between 0 and 6 min. Mobile phase B was increased to 55% from 6 to 6.5 min and held until 10 min. This phase was increased to 100% from 10 to 12 min and held until 16 min. Then, from 16 to 17.5 min, the phase was dropped to 0% and held there until 20 min. Prostanoids were subsequently analyzed using a tandem quadrupole mass spectrometer via multiple-reaction monitoring (MRM) in negative-ion mode. The transitions monitored were m/z: 351/271 for PGE_2_ and PGD_2_, 353/193 for PGF_2α_, 369/207 for 6-ketoPGF_1α_, 369/195 for TXB_2_ and 333/235 for PGB_2_. These prostanoids were identified in samples by matching their MRM signal and LC retention time with those of a pure standard.

### Statistics

Data were expressed as mean ± standard error of the mean (SEM). Data were analyzed by one-way analysis of variance (ANOVA), and then differences among means were analyzed using Tukey-Kramer multiple comparison tests. P-values <0.05 were considered statistically significant.

## Additional Information

**How to cite this article**: Sasaki, Y. *et al.* Genetic-deletion of Cyclooxygenase-2 Downstream Prostacyclin Synthase Suppresses Inflammation Reactions but Facilitates Carcinogenesis, unlike Deletion of Microsomal Prostaglandin E Synthase-1. *Sci. Rep.* **5**, 17376; doi: 10.1038/srep17376 (2015).

## Supplementary Material

Supplementary Information
