# Supplementary material for: Genetic-deletion of Cyclooxygenase-2 Downstream Prostacyclin Synthase Suppresses Inflammatory Reactions but Facilitates Carcinogenesis, unlike Deletion of Microsomal Prostaglandin E Synthase-1
Source: Sci Rep. 2015 Nov 27;5:17376. doi: 10.1038/srep17376 (PMC4661703; doi:10.1038/srep17376)
Supplement: Supplementary Information [file srep17376-s1.pdf]

**Genetic Deletion of Cyclooxygenase-2 Downstream Enzyme Prostacyclin Synthase Suppresses Inflammation but Facilitates Carcinogenesis, unlike Deletion of Microsomal Prostaglandin E Synthase-1**

Yuka Sasaki, Shuhei Kamiyama, Azusa Kamiyama, Konomi Matsumoto, Moe Akatsu, Yoshihito Nakatani, Hiroshi Kuwata, Yukio Ishikawa, Toshiharu Ishii, Chieko Yokoyama, and Shuntaro Hara*

*haras@pharm.showa-u.ac.jp

**Supplementary Table 1.** Composition of peritoneal leukocytes.

| | control | PGIS KO | mPGES-1 KO | DKO |
|---|---|---|---|---|
| Macrophages % | 45.2 ± 4.26 | 34.2 ± 9.79 | 47.6 ± 8.81 | 28.6 ± 7.62 |
| Lymphocytes % | 50.2 ± 3.59 | 60.8 ± 11.9 | 41.4 ± 12.6 | 68.6 ± 7.25 |
| Polynuclear leukocytes % | 4.57 ± 1.59 | 4.92 ± 2.74 | 11.0 ± 3.81 | 2.72 ± 2.40 |

Cytocentrifuge preparations of the exudate leukocytes were Giemsa-stained, and cell subsets were identified and counted. Results are mean±SEM (n=3)
